# Depressive symptoms are associated with leukocyte telomere length in American Indians: findings from the Strong Heart Family Study

**DOI:** 10.18632/aging.101104

**Published:** 2016-11-18

**Authors:** Qi Zhao, Yun Zhu, Fawn Yeh, Jue Lin, Elisa T. Lee, Shelley A. Cole, Darren Calhoun, Jinying Zhao

**Affiliations:** ^1^ Department of Epidemiology, Tulane University School of Public Health and Tropical Medicine, New Orleans, LA 70112, USA; ^2^ Department of Epidemiology, College of Public Health and Health Professions, University of Florida, Gainesville, FL 32611, USA; ^3^ Center for American Indian Health Research, University of Oklahoma Health Science Center, Oklahoma City, OK 73126, USA; ^4^ Department of Biochemistry and Biophysics, University of California San Francisco, San Francisco, CA 94158, USA; ^5^ Department of Genetics, Texas Biomedical Research Institute, San Antonio, TX 78227, USA; ^6^ MedStar Health Research Institute, Phoenix, AZ 85016, USA

**Keywords:** American Indian, depression, leukocyte telomere length, Strong Heart Study

## Abstract

Patients with depression have an increased risk for many aging-related disorders, but the biological mechanisms underlying this link remain to be determined. Here we examined the association between depressive symptoms and leukocyte telomere length (LTL), a marker of biological aging, among 2,175 American Indians participating in the Strong Heart Family Study. Depressive symptoms were assessed by the Center for Epidemiologic Studies of Depression Scale (CES-D), which was categorized into four levels: none (< 10), mild (10-15), moderate (16-24), and severe (> 24). LTL (T/S ratio) was quantified by qPCR. The association between depressive symptoms and LTL was examined by multivariate generalized estimating equation models, adjusting for sociodemographic factors, lifestyle factors, and chronic conditions. Results showed that individuals with a higher level of depressive symptoms had shorter LTL. Specifically, LTL in participants reporting none, mild, moderate, and severe depressive symptoms were 1.000, 0.999, 0.988, and 0.966, respectively (*P* for trend = 0.0278). Moreover, gender appears to modulate the effect of reported depressive symptoms that fall in the severe range (CES-D > 24) on LTL (*P* for interaction = 0.0346). Our results suggest that depressive symptoms may accelerate biological aging through pathways beyond traditional risk factors in American Indians.

## INTRODUCTION

Major depressive disorder (MDD), the second leading cause of disability worldwide, affects about 6.7% of the US population aged 18 years and older in any given calendar year and poses a huge burden on public health and economics [1]. MDD is a major contributor to suicide [2] and has been associated with excess morbidity and mortality [3–5]. Depression also constitutes a significant health problem in the American Indian community [6, 7]. Based on a survey conducted in 2006, American Indians/Alaska Natives experienced a much higher rate of major depressive episodes compared to Caucasians (12.1% vs. 7.8%) [8]. Unraveling the molecular mechanisms underlying depression is the key to lead to novel diagnostic or therapeutic strategies for this debilitating disorder and its co-morbidities.

Telomeres are repetitive DNA sequences on the distal ends of the chromosomes. They are critical in maintaining chromosomal stability during mitotic cell division. Telomere length shortens progressively during each round of cell division and declines significantly with age, thus providing a biomarker for biological aging. Shorter leukocyte telomere length (LTL) has been associated with a wide range of aging-related disorders, such as diabetes, cardiovascular disease, and cancer [9–13]. Among American Indians participating in the Strong Heart Family Study (SHFS), our group has also reported associations of shorter LTL with diabetes [14], carotid atherosclerosis [15], obesity [16], and arterial aging [17].

Previous studies have suggested a strong link between depression, aging and aging-related disorders. For instance, depressed individuals have increased risk for cognitive aging [18], Alzheimer's disease [19], cardiovascular disease [20], diabetes [21], obesity [22], as well as cancer [23]. In addition, a few studies have reported an association of telomere length with depression, although these studies had relatively small sample size and were largely conducted in Caucasian populations [24, 25]. We are not aware of prior studies examining the association between LTL and depression in a sample of American Indians. Given that both LTL and depression are genetically determined, the relationship between depression may be racially/eth-nical specific [26]. In addition, there is support in the literature that women are two times as likely to be diagnosed with depressive disorders than men [27, 28]. However, it is unclear whether the association between depression and telomere length differs by gender. As such, the goals of this study were to examine the relationship between depressive symptoms and LTL, and to test whether gender modulates this association in a group of American Indians, a population that experiences high rates of both depression and aging-related disorders.

## RESULTS

### Characteristics of study participants

Table 1 shows the characteristics of the study participants. Nearly 50% of the study participants reported mild or more severe depressive symptoms with the Center for Epidemiologic Studies of Depression Scale (CES-D) score ≥ 10. Compared to women, men were younger, had lower body mass index (BMI), were physically more active, and more likely to be current drinkers. Moreover, men had lower CES-D scores and a lower rate of antidepressant drug use compared to women. There were no significant differences in the education level and current smoking rate between men and women.

**Table 1:** Characteristics of study participants

	Total (N=2,175)	Men (N=847)	Women (N=1,328)	*P* value*
Age, years	40.4 (17.0)	39.2 (16.9)	41.1 (17.0)	0.0060
Education (high school or higher), %	10.3	9.3	11.0	0.1553
BMI, kg/m^2^	31.3 (7.5)	30.6 (7.0)	31.8 (7.7)	0.0010
Current smoker, %	36.4	36.7	36.1	0.6721
Current drinker, %	58.0	65.7	53.1	< 0.0001
Physical activity, steps/d	5845.3 (3899.9)	7150.9 (4321.4)	5059.3 (3390.0)	< 0.0001
LTL (T/S ratio)	0.994 (0.239)	0.983 (0.237)	1.000 (0.239)	0.0266
CES-D score	12.1 (10.2)	10.3 (8.4)	13.2 (11.0)	< 0.0001
Categories of depressive symptoms				
CES-D < 10 (none), %	50.2	56.6	46.1	< 0.0001
10 ≤ CES-D < 16 (mild), %	21.9	22.3	21.7	
16 ≤ CES-D ≤ 24 (moderate), %	15.6	13.9	16.7	
CES-D > 24 (severe), %	12.3	7.2	15.5	
Antidepressant drug use, %	5.2	1.9	7.2	< 0.0001

Abbreviations: BMI, body mass index; LTL, leukocyte telomere length.

Data are presented as mean (standard deviation) or percentage.

* *P* values for differences between men and women; correlation among family members was corrected by GEE.

### Association between traditional risk factors and LTL

Table 2 presents the age-adjusted associations of several risk factors with LTL. It shows that LTL was significantly and inversely associated with age (*P* < 0.0001) and BMI (*P* < 0.0001). Men had shorter LTL compared to women (*P* = 0.0035). We did not observe associations of education level, smoking, drinking, and physical activity with LTL in our study population.

**Table 2:** Association of LTL with traditional risk factors in American Indians in the SHFS

Variable	β (SE)*	*P* value*
Age	−0.0048 (0.0005)	< 0.0001
Men	−0.0308 (0.0106)	0.0035
Education (high school or higher)	0.0207 (0.0191)	0.2790
BMI	−0.0024 (0.0006)	< 0.0001
Current smoker	0.0020 (0.0085)	0.8121
Current drinker	−0.0155 (0.0114)	0.1723
Physical activity	0.0006 (0.0043)^†^	0.8803

Abbreviations: BMI, body mass index; LTL, leukocyte telomere length.

* Results of multivariate GEE, adjusting for age, sites, male sex, education, body mass index, current smoking, current drinking, and physical activity.

† Associated with 1-standard deviation increase in steps per day.

### Association between depressive symptoms and LTL

Table 3 shows the multivariate-adjusted LTL (95% CI) across the four categories of severity for depressive symptoms. After adjusting for age, gender, and education, individuals with more severe depressive symptoms had a significantly shorter LTL (Model 1: *P* for trend = 0.0322). The association between depressive symptoms and LTL remained statistically significant after further adjustments for lifestyle factors (Model 2: *P* for trend = 0.0356), or anti-depressant uses and chronic conditions (Model 3: *P* for trend = 0.0278).

**Table 3:** Multivariate association between LTL and severity of depressive symptoms in American Indians in the SHFS (N=2,175)

Model*	Multivariate-adjusted LTL (95% CI)				*P* for trend
	None CES-D < 10 (N=1,091)	Mild 10 ≤ CES-D < 16 (N=477)	Moderate 16 ≤ CES-D ≤ 24 (N=340)	Severe CES-D > 24 (N=267)	
Model 1: Age, gender, site, and education	0.974 (0.948, 1.000)	0.964 (0.937, 0.991)	0.963 (0.932,0.995)	0.942 (0.912,0.973)	0.0322
Model 2: Age, gender, site, education, BMI, smoking, drinking, and physical activity	0.987 (0.962, 1.012)	0.986 (0.959, 1.013)	0.976 (0.943, 1.009)	0.955 (0.925, 0.986)	0.0356
Model 3: Age, gender, site, education, BMI, smoking, drinking, physical activity, use of antidepressants, and presence/absence of chronic diseases	1.000 (0.967, 1.033)	0.999 (0.965, 1.032)	0.988 (0.957, 1.020)	0.966 (0.932, 1.001)	0.0278

Abbreviations: BMI, body mass index; LTL, leukocyte telomere length.

* Correlations among family members was adjusted in all three GEE models.

### Gender difference in the association between depressive symptoms and LTL

**Figure 1:**
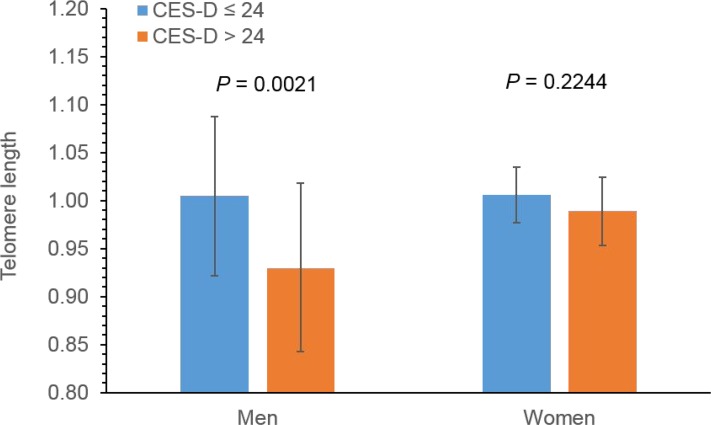
Sex-specific effect of severe depressive symptoms on LTL in American Indians *P*-values were adjusted for age, site, education, BMI, smoking, drinking, physical activity, use of antidepressants chronic conditions, study sites using GEE.

We identified a significant interactive effect between gender and severe depressive symptoms on LTL (Table 4). Even after adjusting for all covariates, men with severe depressive symptoms (CES-D > 24) had significant shorter LTL than those without (CES-D ≤ 24), whereas no significant association was found in women (Figure 1). In a fully adjusted model (Model 3), LTL were 1.0048 (95% CI: 0.9224, 1.0872) and 0.9301 (95% CI: 0.8425, 1.0178) for men with and without severe depressive symptoms, respectively (*P* = 0.0021). In women, LTL were 1.0059 (95% CI: 0.9224, 1.0872) and 0.9886 (95% CI: 0.9534, 1.0237) for individuals with and without severe depressive symptoms, respectively (*P* = 0.2244).

**Table 4:** Gender-specific association between LTL and severe depressive symptoms (CES-D > 24)

Model*	Men		Women		*P* value for interaction between gender and severe depressive symptoms
	β (SE)	*P* value	β (SE)	*P* value	
Model 1: Age, site, and education	−0.0653 (0.0210)	0.0019	−0.0147 (0.0138)	0.2872	0.0469
Model 2: Age, site, education, BMI, smoking, drinking, and physical activity	−0.0724 (0.0245)	0.0031	−0.0168 (0.0142)	0.2375	0.0329
Model 3: Age, site, education, BMI, smoking, drinking, physical activity, use of antidepressants, and presence/absence of chronic diseases	−0.0746 0.0243)	0.0021	−0.0173 (0.0142)	0.2244	0.0346

Abbreviations: BMI, body mass index; LTL, leukocyte telomere length.

* Correlations among family members was adjusted in all three GEE models.

## DISCUSSION

This study provides initial evidence for an association between depressive symptoms and LTL in a large sample of geographically diverse American Indians. We found that a higher level of depressive symptoms was significantly associated with shorter LTL, independent of many known risk factors. Moreover, gender appears to modulate the relationship between severe depressive symptoms and LTL. Specifically, severe depressive symptoms were associated with shortened LTL in men, but not in women.

Several previous studies have investigated the relationship between MDD and LTL [24, 25]. For example, MDD was found to be significantly associated with shortened telomere length in a study comprising 15 MDD patients and 44 nondepressed controls [29], but this association was unable to be replicated in another cross-sectional study comprising 1,164 participants [30]. The relationship between depressive symptoms and LTL was also reported in other studies. However, those studies were largely conducted in Caucasians and results were inconsistent [24, 25]. Given that both LTL and MDD are under genetic control [26], results identified in Caucasians are unlikely to be generalized to American Indians. American Indians may have different risk profiles from the Caucasian population. To date, little is known about the relationship between depression and telomeres in American Indians. In a large sample of American Indians, here we provide initial evidence that is supporting a relationship between depressive symptoms and accelerated cellular aging. Moreover, there appears a dose-response relationship between the severity of depressive symptoms and LTL, with participants with more severe depressive symptoms exhibiting shorter LTL in our study population.

While the precise mechanisms linking depression to LTL shortening remain to be determined, it is possible that depression could contribute to shortened LTL through one or more biological pathways known to be involved in depression, such as inflammation [31, 32], oxidative stress [33, 34], hyperactivity of the hypothalamic-pituitary-adrenal axis [35, 36], and increased activity of the autonomic nervous system [37, 38]. It has been demonstrated that depression is accompanied by oxidative stress dysregulation, including increased levels of free radicals and oxidative damage products as well as decreased levels of antioxidants [34]. Meanwhile, oxidative stress could induce the formation of 8-oxodG at the GGG triplet in telomere sequence. Human 8-oxodG-DNA glycosylase introduces a chain break in a double-stranded oligonucleotide specifically at an 8-oxodG residue, leading to telomere shortening [39]. These lines of evidence support the hypothesis that aforementioned dysregulations accumulated in depression may mediate its effect on telomere attrition.

Our analysis revealed a gender difference in the association between depressive symptoms and shortened LTL. The effect of severe depressive symptoms on LTL was much stronger in men than in women. This finding appears to be consistent with a recent study showing that depression was associated with LTL in men, but not in women [40]. Despite the fact that women are more likely to be depressed than men, there has been evidence that men with mental disorders tend to have worse outcomes than women [41–43]. For example, in a cohort of Netherlands, men with minor depression defined by CES-D ≥ 16 had a 1.8-fold higher risk of death during follow-up than non-depressed men. However, minor depression did not significantly increase mortality risk in women [41]. According to a national survey of the US, the hazard ratios for all-cause mortality associated with major depression were 3.1 and 1.7 for men and women, respectively [42]. The mechanisms underlying this gender difference are unknown. It is possible that estrogens may play a role in these observed gender differences because they have been implicated in increasing the activity of telomerase [44]. Further research is needed to confirm this gender-specific association and to elucidate the potential role of estrogens in the association.

The major strengths of our study include a large sample size, the high-quality telomere and clinical data, as well as the sophisticated statistical models with extensive adjustments of known covariables. However, some limitations of this study should also be discussed. First, as all cross-sectional studies, the causal relationship between depressive symptoms and shortened telomere length could not be determined in our analysis. Second, due to lack of fresh blood samples, we did not measure the activity of telomerase, which may mediate the effect of depressive symptoms on telomere length. Third, as our study participants were exclusively American Indians, our results may not be generalized to other ethnic groups. And lastly, although our sample size in this study is considerable, American Indians are a very geographically and culturally diverse group of people. In the US, there are over 550 recognized tribal groups; therefore this study may also be limited in its applicability or generalization to American Indians throughout the population of the US.

Nonetheless, in this large epidemiological study of American Indians, we provide the first evidence that depressive symptoms were associated with shorter LTL, independent of many known risk factors. The effect of severe depressive symptoms on accelerated telomere shortening could be more prominent in men than in women. These findings need to be replicated in large-scale populations of different races/ethnicities. Longitudinal studies are warranted to establish the causal relationship between depression and telomere erosion in future investigations.

## METHODS

### Study subjects

The current study included a total of 2,175 American Indians (847 men and 1,328 women) participating in the SHFS, a family-based prospective study of genetic, metabolic, and behavioral factors for cardiovascular disease (CVD), diabetes, and their risk factors. The SHFS participants were recruited from 12 tribes residing in Arizona, Oklahoma, and South/North Dakota in 2001-2003. A detailed description of the study design and methods of the SHFS has been reported previously [45]. In brief, information on demographic factors, socioeconomic status, lifestyle factors, medical history, and medication use was collected by personal interview using standard questionnaires. A physical examination was conducted and fasting blood samples were collected for laboratory tests. The SHFS study protocol was approved by the Institutional Reviews Boards from the Indian Health Service and the participating centers. All subjects gave informed consent.

### LTL measurement

Genomic DNA from peripheral blood was isolated according to standard methods. The LTL measurements were performed by Dr. Blackburn's laboratory at the University of California, San Francisco using a high-throughput telomere length assay system. The LTL assay determines the ratio of telomeric product (T) to a single copy gene (S) obtained using quantitative polymerase chain reaction (qPCR) according to protocols described previously [46, 47]. The rationale of this method is that the longer the telomeres are in each sample, the more PCR product will be generated in PCR reactions using primers specific for the telomeric DNA. The T/S ratio reflects the average length of the telomeres. For quality control, seven control DNA samples from various cancer cell lines were included in each assay plate. These control samples allowed us to create standard curves, which were then integrated into a composite standard curve used for T and S concentration calculations. In addition, 4.1% of the total sample was assayed in duplicate. Telomere length of the replicate samples were highly correlated (r = 0.95, *P* < 0.0001). Lab technicians were blinded to any knowledge of clinical data. In our analysis, the intra-assay coefficient of variation was 4.6% and the inter-assay coefficient of variation (assay-to-assay) was 6.9%.

### Assessment of depressive symptoms

In the SHFS, we assessed depressive symptoms using the CES-D, which comprised of 20 items and was administered as a self-report instrument [48]. The CES-D has been widely used in large-scale epidemiological studies, such as the Honolulu Heart Program, the Inter-Tribal Heart Project, Coronary Artery Risk Development in Young Adults, and the Stanford Coronary Prevention Project [49]. It has also been used in studies including American Indians with good internal consistency [50–52].

The CES-D was designed to measure the current level of depressive symptoms [48]. The 20 items represent all major components of depressive symptoms, including depressed mood, feeling of guilt and worthlessness, feeling of helplessness and hopelessness, loss of appetite, sleep disturbance, and psychomotor retardation. The 20 items were rated on a four-point Likert scale, ranging from “rarely, or not at all,” scored as 0, to “most of the time,” scored as 3. Four positively worded items were reversed when scored. Item scores were then summed for a total depression score with higher scores indicating increased reported depressive symptoms. Depressive symptoms were categorized into four levels: none (CES-D < 10), mild (10 ≤ CES-D < 16), moderate (16 ≤ CES-D ≤ 24), and severe (CES-D > 24). These cut-offs have been widely used in previous epidemiological studies [48, 49].

### Assessments of covariates

BMI was calculated as weight divided by squared height (kg/m^2^). Current smokers were defined as who reported smoking 100 or more cigarettes in their lifetime and were currently smoking every day or some days. Current drinkers were those who had consumed any alcohol during the past year [53]. The level of physical activity was assessed by the average steps per day during a 7-day step recording by a pedometer. Information on diagnosis and treatment of CVD, diabetes, hypertension, and chronic kidney disease was also collected.

### Statistical analyses

To examine the association between depressive symptoms and LTL, we constructed multivariate hierarchical generalized estimating equation (GEE) models in which continuous LTL (T/S ratio) was the dependent variable and severity of depressive symptoms (categorized into none, mild, moderate, severe) was the independent variable: Model 1, adjusted for socio-demographic factors (age, gender, and education); Model 2, additionally adjusted for lifestyle factors (BMI, smoking, drinking, and physical activity); and Model 3, further adjusted for antidepressant use (yes/no), and presence or absence of chronic conditions (CVD, diabetes, hypertension, and chronic kidney disease). The GEE model was used to account for the correlations among family members. To examine whether gender modulates the association between depressive symptoms and LTL, we included an interaction term of gender and the categorical variable for depressive symptoms in the GEE models. Gender-stratified analyses were also conducted if any interaction term was significant. All analyses were performed using SAS version 9.3 (SAS Institute, Cary, NC).
